# Modulation of Human Valve Interstitial Cell Phenotype and Function Using a Fibroblast Growth Factor 2 Formulation

**DOI:** 10.1371/journal.pone.0127844

**Published:** 2015-06-04

**Authors:** Najma Latif, Alfred Quillon, Padmini Sarathchandra, Ann McCormack, Alec Lozanoski, Magdi H. Yacoub, Adrian H. Chester

**Affiliations:** 1 Imperial College, Heart Science Centre, Harefield Hospital, Harefield, Middx, UB9 6JH, United Kingdom; 2 QCRC, Qatar Cardiovascular Research Centre, Qatar Foundation, Doha, Qatar; 3 Département de Biologie, École Normale Supérieure de Lyon, Université de Lyon, UCB Lyon1, 46 Allée d’Italie, Lyon, France; Centro Cardiologico Monzino, ITALY

## Abstract

Valve interstitial cells (VICs) are fibroblastic in nature however in culture it is widely accepted that they differentiate into a myofibroblastic phenotype. This study assessed a fibroblast culture media formulation for its ability to maintain the phenotype and function of VICs as in the intact healthy valve. Normal human VICs were cultured separately in standard DMEM and in fibroblast media consisting of FGF2 (10ng/ml), insulin (50ng/ml) and 2% FCS for at least a week. Cell morphology, aspect ratio, size, levels and distribution of protein expression, proliferation, cell cycle, contraction and migration were assessed. Some VICs and some valve endothelial cells expressed FGF2 in valve tissue and this expression was increased in calcified valves. VICs in DMEM exhibited large, spread cells whereas VICs in fibroblast media were smaller, elongated and spindly. Aspect ratio and size were both significantly higher in DMEM (p<0.01). The level of expression of α-SMA was significantly reduced in fibroblast media at day 2 after isolation (p<0.01) and the expression of α-SMA, SM22 and EDA-fibronectin was significantly reduced in fibroblast media at days 7 and 12 post-isolation (p<0.01). Expression of cytoskeletal proteins, bone marker proteins and extracellular matrix proteins was reduced in fibroblast media. Proliferation of VICs in fibroblast media was significantly reduced at weeks 1 (p<0.05) and 2 (p<0.01). Collagen gel contraction was significantly reduced in fibroblast media (p<0.05). VICs were found to have significantly fewer and smaller focal adhesions in fibroblast media (p<0.01) with significantly fewer supermature focal adhesions in fibroblast media (p<0.001). Ultrastructurally, VICs in fibroblast media resembled native VICs from intact valves. VICs in fibroblast media demonstrated a slower migratory ability after wounding at 72 hours (p<0.01). Treatment of human VICs with this fibroblast media formulation has the ability to maintain and to dedifferentiate the VICs back to a fibroblastic phenotype with phenotypic and functional characteristics ascribed to cells in the intact valve. This methodology is fundamental in the study of normal valve biology, pathology and in the field of tissue engineering.

## Introduction

Heart valves are living structures whose cells play a fundamental role in the function, durability and longevity or the valve [1]. The presence of viable cells allows the aortic valve to perform a complex repertoire of functions that serve to preserve the unidirectional flow of blood out of the left ventricle, optimise coronary blood flow and preserve myocardial function. The valve is comprised of extracellular matrix, on which reside a population of valve endothelial cells lining both surfaces of the valves. The body of the matrix is populated by interstitial cells (VICs) that are dispersed throughout the three distinct layers of the valve cusps. VICs have been ascribed a fibroblastic phenotype due to the absence of specific markers of other cell types and possess a wide range of biological properties that distinguishes them from other fibroblast-like cells and allows them to contribute to maintaining valve function [1]. Their morphology by electron microscopy such that they are mostly flattened cells lacking a basement membrane and extend multiple processes and due to their ability to synthesize extracellular matrix proteins and matrix-degrading enzymes which include matrix metalloproteinases and their inhibitors (TIMPs) respectively. Their principle function is to remodel the matrix for homeostasis and during adaptation during disease pathogenesis. In healthy adults, VICs are predominantly quiescent fibroblasts with a small population of smooth muscle cells which reside in the base of the ventricularis[2]. It has been reported that myofibroblasts are consistently present in aortic valve leaflets[3] however it was not stipulated what proportion of total cells this comprised. We believe that the number of myofibroblasts in normal aortic valve leaflets is extremely low (<1%)[2].

During the developmental process of valve morphogenesis, the valve leaflets arise from the endocardial cushions and a subpopulation of endocardial cells differentiate through a process of endothelial-to-mesenchymal transformation into valvular cells[4]. These fetal valvular cells express α-smooth muscle actin (α-SMA) and are regarded as activated myofibroblasts[5]. The VICs soon lose this expression of α-SMA after birth[6].

In vivo, transmission electron microscopy of VICs has shown classical features of fibroblasts with long cytoplasmic extensions, prominent adhesion and gap junctions and a close association with the extracellular matrix[7]. Adherens junctions were prominent and occasional gap junctions were identified. The cells demonstrated a rich array of intermediate filaments, varying amounts of endoplasmic reticulum and Golgi and few prominent stress fibers. Due to their plasticity, the VIC population consists of a number of different phenotypic states which include quiescent, activated, progenitor and osteoblastic cells which may co-exist under various physiological and pathophysiological conditions[8]. VICs have been shown to become re-activated to a myofibroblastic phenotype with the neo-expression of α-SMA during aging[5], valve sclerosis and stenosis[5], mechanical damage[9] and in tissue engineered valves[5]. However, when these cells are isolated and maintained in culture, within a very short time 50–78% of VICs become α-SMA-positive and thus emulate a fetal/pathological VIC[10]. VICs in culture have demonstrated two different morphologies; cuboidal and spindly, and notably, 100% of porcine VICs in culture stained positive for α-SMA[7]. Della Rocca *et al* showed that 3 cell types exist in the aortic and pulmonary valves; smooth muscle cells, myofibroblasts and fibroblasts[11], the former two cell types showing restricted expression to the fibrosa and Messier *et al* concluded that aortic VICs were best designated as myofibroblasts[12]. We have previously shown that most of the VICs in the native aortic valves do not express α-SMA[2] and that a proportion of normal, healthy, human VICs in culture express α-SMA[10, 13]. Porcine VICs in culture also express α-SMA[7] and by passage 3–5, most if not all porcine VICs express α-SMA and are of a myofibroblastic nature (our unpublished findings).

There is consensus that the majority of VICs are fibroblastic in vivo, however in culture they are a heterogeneous population[13, 14] with mixed phenotypes. It has also been suggested that cultured VICs possess characteristics similar to smooth muscle cells[15, 16] as the different valves harbour differing numbers of smooth muscle cells. This in vitro population does not emulate the native VIC population as it is generally accepted that most fibroblasts isolated from many different organs in culture differentiate into a myofibroblastic phenotype with the expression of α-SMA[17].

FGF2 is a member of the fibroblast growth factor family (FGF) which includes 23 members. It is expressed in almost all tissues and plays important roles in normal and pathological processes. FGF2 binds to a family of four distinct, high affinity tyrosine kinase receptors, designated FGFR-1 to -4 [18]. It also binds to the extra-cellular matrix and heparan sulphate is an essential and dynamic regulator of FGF signalling. FGF2 has been previously shown to be expressed by porcine VICs in culture[19] and treatment with FGF2 was shown to repress myofibroblast activation in porcine VICs [20]. FGF2 also has been shown to promote VIC repair[19, 21, 22]. The role of FGF2 has not been investigated in normal human VICs. We hypothesised that reducing the serum content of the media will also reduce the concentration of activating growth factors such as TGFβ, and to promote proliferation, insulin was added as a mitogenic factor[23–25]. Hence, we have subsequently used a formulation of media containing FGF2/low serum/insulin to assess the phenotypic and functional effects which could decrease this myofibroblastic differentiation in vitro of the native human VIC back to more of a fibroblastic phenotype which would provide a better model to study valve biology and pathogenesis.

## Materials and Methods

18 normal, tricuspid, aortic valves, free from calcification (mean age 48.5 years, 13 male: 5 female; age range 6–58 years; SD = 12.47) were used in this study. The normal valves were obtained from patients free from cardiovascular and valvular complications based on history, macroscopic and microscopic evaluation. These were unused valves from healthy, heart donors, most of whom died from a cerebral haemorrhage due to head trauma with no underlying diseases. The ischaemic time for fresh tissue harvest did not exceed 24 hours. Left ventricular dysfunction was not considered a contraindication however patients with advanced cancer or other debilitating diseases with a life expectancy of less than 6 months were excluded. 5 calcified valves were used (mean age 58 years; all male; age range 41–66 years; SD = 8.4).

All human studies have been approved by the Brompton and Harefield trust ethics committee and have therefore been performed in accordance with the ethical standards laid down in the 1964 Declaration of Helsinki and its later amendments. All persons gave their written informed consent prior to their inclusion in the study.

### Valve Cell Isolation and Culture

Normal valve leaflets were excised, washed in Phosphate Buffer Saline 1X (PBS, Sigma) and incubated in a collagenase solution (type A, 0.15% w/v, Roche) under a forceful agitation for 10 minutes at 37°C to remove the endothelial cells. The undigested tissue was removed, washed in PBS and digested for a further 50 minutes to isolate the VICs. The VIC suspension was centrifuged and the resulting pellet resuspended in tissue culture medium and plated out in tissue culture flasks.

Two media were used for our study: a “classical DMEM” containing basal Dulbecco's Modified Eagle Medium (DMEM, Sigma), 150U/ml penicillin/streptomycin (Sigma), 2mM L-glutamine (Sigma) and 10% heat-inactivated fetal calf serum (FCS) (Helena Biosciences, Sunderland, UK) and used in a large number of previous studies to maintain VICs in culture[2, 26–28]; and a “fibroblast media” defined as a 2% FCS in DMEM containing 150U/ml penicillin/streptomycin (Sigma), 2mM L-glutamine (Sigma) and supplemented with 50ng/ml insulin and 10ng/ml fibroblast growth factor-2 (FGF2, Sigma). The VICs were grown in classical DMEM until confluent and subsequently divided between the two media and cultured for a further 14 days before analysis. VICs were also cultured directly in fibroblast media and used for analysis. VICs from different heart valves were used separately between passages 3–6.

### Immunostaining

Valves were fixed in 10% formal saline for 24 hours, washed in distilled water and immersed in EDTA for 2 weeks at 37°C after which processing for paraffin sections was carried out. Sections were washed twice in PBS and blocked using 3% bovine serum albumin (w/v) (BSA) in PBS containing 1% v/v Tween-20. Sections were incubated separately for 1hour with antibody against FGF2. Negative control consisted of 3% BSA in PBS containing 1% v/v Tween 20, isotype controls for the monoclonals and rabbit serum for the polyclonals. Primary antibody was then removed by washing the sections 3 times in PBS followed by a second layer of biotinylated goat anti-mouse (GAM IgG-Vector laboratories) in PBS. Sections were then washed 3 times in PBS before 1 hour incubation with Avidin-Biotin Complex ABC-Vector laboratories). Reactivity was detected using diaminobenzidine tetrahydrochloride (DAB tablets- Sigma) (25mg/ml) and hydrogen peroxide (0.01% W/V). Sections were then counter stained with Mayers haematoxylin and viewed on Ziess LSM 510 confocal microscope.

VICs were seeded on coverslips and cultured for 14 days. The coverslips were washed two times in PBS and fixed in 4% paraformaldehyde for 10 minutes. The fixative solution was removed with three rinses with PBS, cells were permeabilised with Triton X-100 (0.5% v/v in PBS) for 3 minutes and washed two times in PBS-Tween (PBS-T, 0.1% v/v). Coverslips were blocked using 3% (w/v) bovine serum albumin (BSA) and incubated with primary antibodies (α-SMA, vimentin, calponin (Dako); vinculin phalloidin fibronectin EDA-fibronectin, collagens, FSA (Dianova), cbfa, osteonectin, osteopontin, SM22 (Abcam), paxillin (sigma), MRTF-A (Santa-Cruz) in BSA 1.5% w/v for one hour. After thorough washing in PBS-T, the coverslips were incubated with secondary antibodies (Table 1) for one hour, washed 3 times during 5 minutes in PBS-T and incubated 10 minutes with 4,6-diamidino-2-phenylindole (DAPI, Sigma). Coverslips were washed again 2 times in PBS-T and mounted on glass slides in Permafluor aqueous mounting fluid (Beckman Coulter, Fullerton, CA). Observations were performed with confocal imaging technology (Zeiss, LSM 510 Meta inverted).

**Table 1:** Median percentages and inter-quartile ranges of VICs positive for α-SMA, SM22 and EDA-fibronectin at 2, 7 and 12 days after isolation and culture in DMEM and fibroblast media.

	α-SMA		SM22		EDA-FIB	
	DMEM	FIB	DMEM	FIB	DMEM	FIB
**DAY 2**	**25 (20–35)**	**8 (3.5–9)** *	**19 (12.5–26)**	**12 (9–27.5)** *	**10 (8.55–14.5)**	**8 (6–10)** *
**DAY 7**	**60 (50–69)**	**20 (6–29)**	**78 (70–89.5)**	**26 (18–33.5)** *	**73 (58–89.5)**	**18 (15–27)** *
**DAY 12**	**83 (76–87.5)**	**26 (21–32)**	**92 (76–87.5)**	**30 (25–38.5)** *	**92 (87–96.5)**	**25 (21.5–32)** #

DMEM vs FIB

* P<0.01

^#^p<0.05

### Western Blotting

VICs were washed two times in PBS, solubilised and homogenised in RIPA buffer solution (Sigma) supplemented with protease inhibitor cocktail 1X (Roche). Cells were scraped with a rubber policeman; the lysate was transferred into a 1.5ml microtube and vortexed. Proteins were quantified with a Pierce BCA protein assay (Thermo Scientific) after a 10,000g centrifugation for 10 minutes at 4°C. Total protein homogenates (7.5 μg) were denatured and separated on 10% Bis-Tris gels (Invitrogen). Electrophoretically resolved bands were then transferred on nitrocellulose membranes (Hybond C, Amersham). Membranes were blocked for 1 hour in Phosphate-Buffered Saline (PBS) containing 0.1% Tween-20 (PBS-T) and 5% (w/v) non-fat powdered milk. Then, they were incubated for 1 hour with primary antibodies (Table 1) in PBS-T containing 5% (w/v) non-fat powdered milk. Membranes were then washed three times in PBS-T and incubated with corresponding horseradish peroxidase conjugate secondary antibody (Table 1) for 1 h at room temperature in PBS-T. Membranes were washed five times in PBS-T. Visualization of the protein bands was accomplished using enhanced chemiluminescence (ECL) substrate (Amersham) and positivity was captured on Hyperfilm (Amersham). Films were scanned and bands were quantitated using the QuantityOne program (Biorad). Levels of expression were normalised to GAPDH.

### Proliferation assay

2000 cells per well were plated in a 96 well plate and cultured for one and two weeks in different media under normal conditions. Proliferation assay was carried out with CellTiter 96 AQueous Non-Radioactive Cell Proliferation Assay kit (Promega G-5421) by adding 20μL of MTS/PMS solution with 100μL of DMEM on cells. Plate was incubated one hour at 37°C, 5% CO_2_ and absorbance was read at 490nm.

### Flow Cytometry

VICs were counted and 10^5^ were permeabilized in cold 70% ethanol in PBS 2mM EDTA for 30 min on ice. Cells were centrifuged, washed one time in PBS/EDTA and incubated for 30 min at obscurity into 1ml containing 40μg/ml propidium iodide (Sigma), 200μg/ml RNAse A (Sigma), 0.1% triton X-100 (v/v, Sigma) in PBS EDTA. Cells were washed once and the pellet was resuspended in 500μl of PBS/EDTA. 10,000 events were acquired on an EPICS XL flow cytometer (Beckman Coulter).

### Contraction Assay

80% PureColl gel was mixed with 10% PBS (x10) and pH was adjusted to7.4. VICs were counted and 1x10^5^ cells were mixed with the Purecoll gel in a 1:3 ratio in a well of a 96-well plate and allowed to polymerise in an incubator. After 2 hours, additional media was added and the gel dislodged from the well with a needle. It was allowed to contract over an 18 hour period.

### Electron microscopy

Pieces of tissue (2mm^2^) excised from normal valves and cells cultured in both media were fixed in 3% glutaraldehyde (Agar Scientific Ltd., Essex, UK) in 0.1 M phosphate buffer for 2 h. After two buffer washes, the secondary fixation with 1% osmium tetroxide (Agar Scientific Ltd.) in 0.1 M phosphate buffer was carried out for 1 h at room temperature. The specimen was then dehydrated through an increasing ethanol series, starting from 25% ethanol. After dehydration, the tissue was transferred into Eppendorf tubes containing propylene oxide (Sigma-Aldrich, UK). The tissue was then infiltrated with 1:1 propylene oxide:Araldite CY212 resin overnight. After two changes of fresh resin, for a minimum of 3 h each, the tissue was embedded in Araldite CY212 resin and polymerized at 60°C for 18 h. Ultra-thin sections were cut using a Diatome diamond knife on a Reichert-Jung Ultracut E ultramicrotome, floated onto distilled water, collected on copper grids, and stained with 2% uranyl acetate and lead citrate for 10 min in each solution. The stained sections were viewed on a Phillips CM12 electron microscope. Cells were surveyed in the spongiosa and fibrosa of calcified valves adjacent to the region of calcification. 5 regions of each calcified valve were analysed and each area was 200um^2^ in size.

### Migration Assay

VICs cultured in DMEM and fibroblast media were plated on fibronectin (10ug/ml) coated plates overnight before wounding using a 1000μl pipette tip. The distance of the wound was recorded at multiple points along the wound at time 0 and at various intervals over 72hours.

### Statistics

Experiments were run in triplicates with 3–7 samples per assay. Cell dimensions and focal adhesions were measured using ImageJ. Values are given as medians and error expressed by giving the Interquartile Range (IQR). The data was assessed for normality and t-tests and Mann Whitney U tests were applied accordingly. Significance was set at p<0.05.

## Results

### VICs express FGF2 in situ and demonstrate distinct morphologies in DMEM compared to fibroblast media

**Fig 1:**
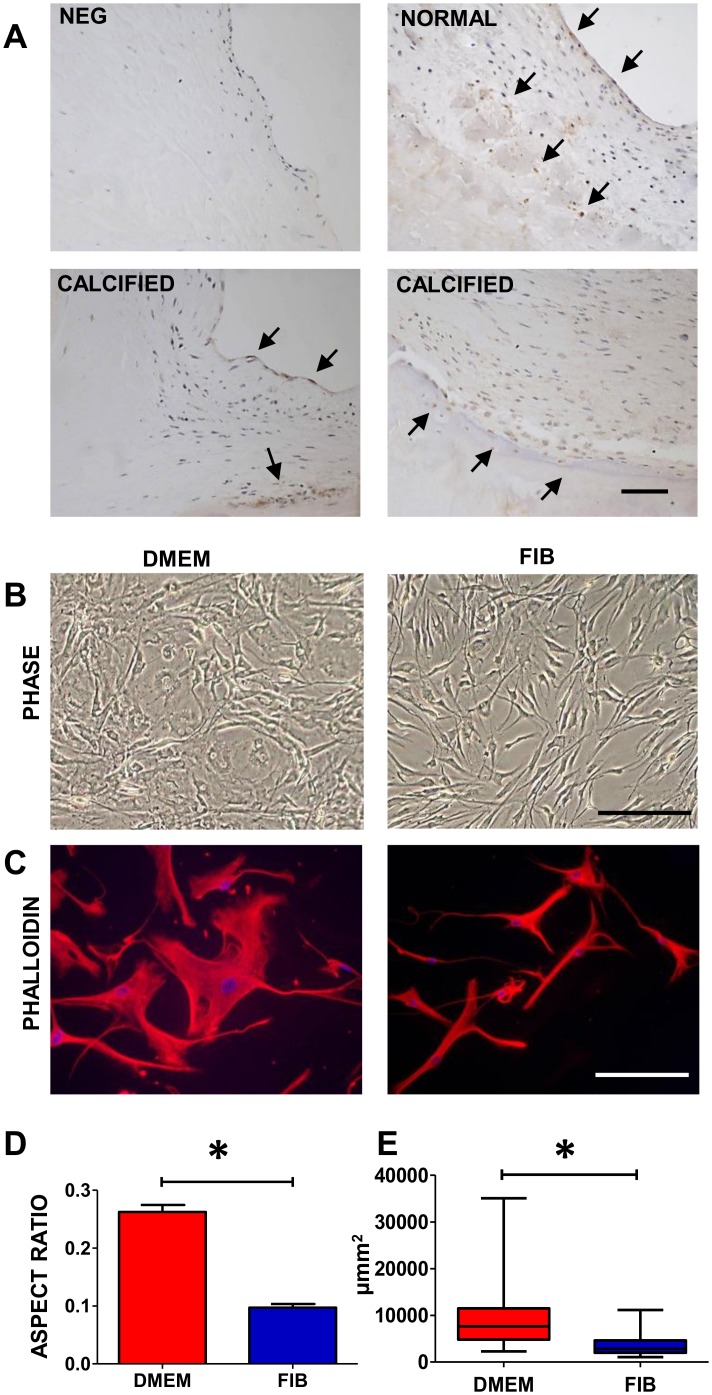
Top panels show a negative control and immunostaining with FGF2 antibody in normal aortic valve leaflets and bottom panels show expression of FGF2 in calcified valves (A); phase contrast images of VICs in DMEM and fibroblast media (B); staining with phalloidin (C), and graphs showing the aspect ratio and cell size (D). Data labelled as FIB is from VICs that were switched to fibroblast media for 2 weeks prior to analysis. Scale bars represent 200μm in A, 50μm in B and 100μm in C. * P< 0.001.

Sections of normal valve leaflets and calcified leaflets were stained with FGF2 antibody and some, not all, valve endothelial cells and VICs demonstrated the expression of FGF2 (Fig 1A). 3 of the 5 calcified leaflets showed regions of leaflets with stronger staining of both the endothelial cells and the VICs. This was most prominent adjacent to the calcified regions.

VICs grown in classical DMEM media showed the typical spread morphology when viewed under phase contrast microscopy and tended to grow over each other (Fig 1B). However VICs grown in fibroblast media were visibly different in morphology such that they were classically spindle shaped with long extensions and thin central bodies. This difference in morphology was clearly apparent when stained using phalloidin with the VICs in DMEM appearing bigger and wider in size and the VICs in fibroblast media being thin and compact (Fig 1C). Cells in both media demonstrated long extensions however these were thinner in fibroblast media. There was a significant difference (p<0.001) in the aspect ratio of the VICs in the different media with the VICs in DMEM being rhomboid and rounded (mean aspect ratio = 0.26;) and the VICs in fibroblast media being longer and thinner (mean aspect ratio = 0.09;) (Fig 1D). There was also a significant difference in the overall size of the VICs in the two different media (VICs in DMEM being a median size of 7636 μm^2^; IQR = 4814–11491 and the VICs in fibroblast media being 2852 μm^2^ in size; IQR = 1995–4368) (p<0.001).

### Time course of VIC activation in DMEM

**Fig 2:**
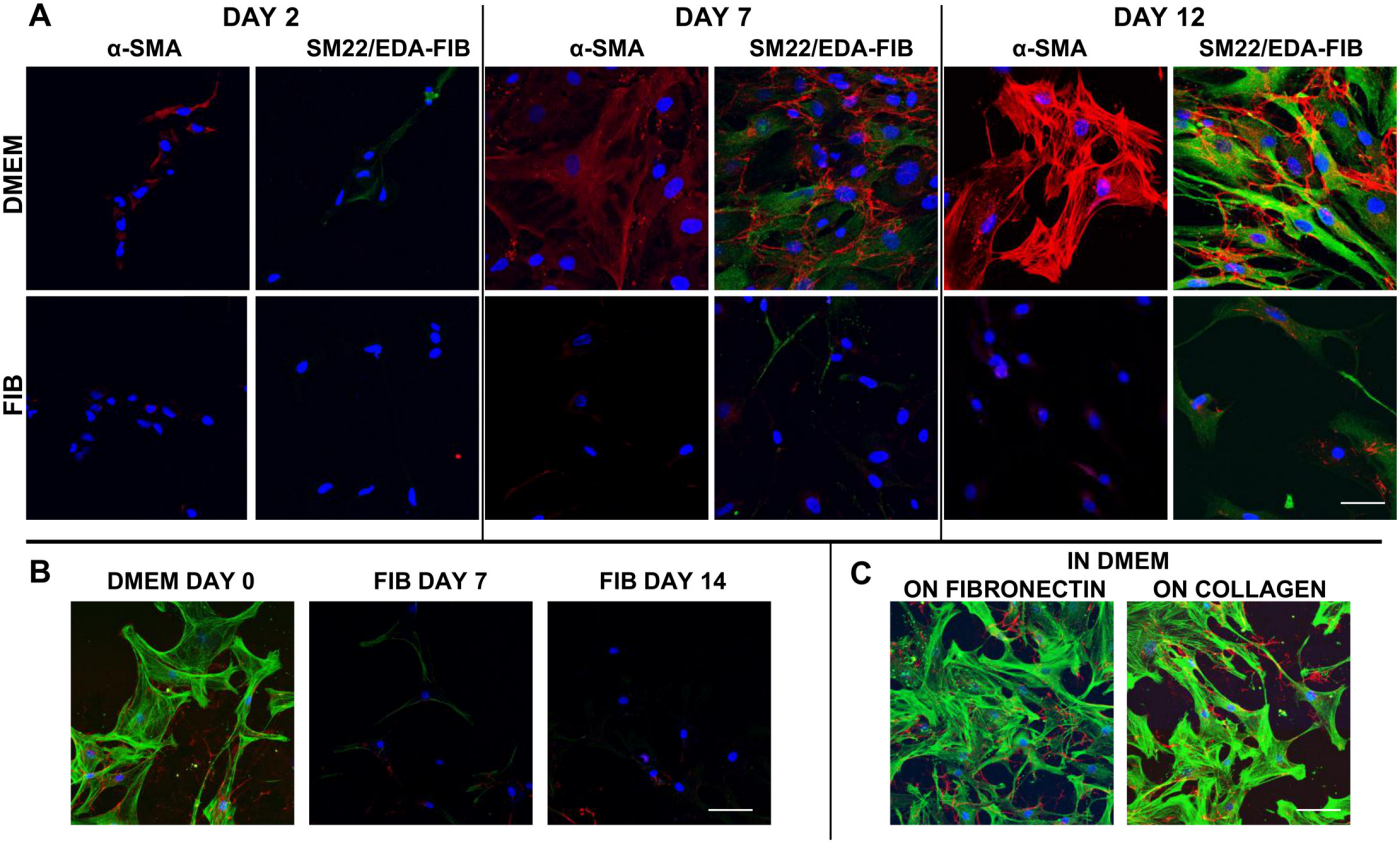
Confocal microscopy of cultured VICs in DMEM and FIB stained with α-SMA and doubly stained with SM22 (green) and EDA-fibronectin (red) at 2, 7 and 12 days after isolation (A). VICs in these panels were cultured in DMEM and FIB from the point of isolation. B shows dedifferentiation of VICs from DMEM at day 0, switched to fibroblast media for 7 days (FIB day 7) and continued in fibroblast media for 14 days (FIB day 14). α-SMA is green and EDA-fibronectin is in red in panels B and C. Scale bar represents 20μm in A and 50μm in B and C.

Freshly isolated primary VICs were plated on coverslips in DMEM and fibroblast media separately and fixed over time intervals to assess the level of activation of VICs (Fig 2A). 2 days after culture, VICs showed clear signs of activation and myofibroblastic differentiation with diffuse expression of α-SMA, SM22 and EDA-fibronectin in DMEM. In fibroblast media at day 2, significantly fewer VICs showed weaker expression of SM22 and EDA-fibronectin and barely detectable levels of α-SMA in a median of 8% of VICs (Table 1). At 7 days post isolation, over 60% of VICs in DMEM demonstrated expression of these markers with expression of α-SMA and SM22 being fibrillar and less than 26% of VICs in fibroblast media showing their expression which was weaker in intensity and remaining diffuse. EDA-fibronectin was clearly expressed and deposited around the cells in DMEM. Significantly more VICs in DMEM demonstrated the expression of these markers (p<0.01). The VICs in DMEM were considerably larger in DMEM at 7 days post isolation whereas the VICs in fibroblast media were elongated and spindly. By 12 days post isolation, the VICs in DMEM were larger and over 83% showed strong fibrillar staining for α-SMA, SM22 and EDA-fibronectin whereas in fibroblast media, they retained their spindly morphology and fewer VICs expressed these markers with markedly reduced intensity.

Flow cytometric analysis was carried out on VICs in DMEM and fibroblast media and the mean fluorescence intensity of α-SMA, EDA-fibronectin and calponin was significantly reduced in fibroblast media Table 2. The antibody against SM22 was unsuitable for FACS.

**Table 2:** Showing flow cytometric data for VICs expressing markers in DMEM and fibroblast media.

MARKER	MEAN FLUORESCENCE INTENSITY		P VALUE
	DMEM MEDIA	FIB MEDIA	
α-SMOOTH MUSCLE ACTIN	24.90	16.64	0.014*
EDA-FIBRONECTIN	11.25	9.57	0.027*
CALPONIN	6.00	2.85	0.003*
VIMENTIN	8.46	8.79	0.82

* p<0.05.

VIC myofibroblasts could be dedifferentiated by incubating in fibroblast media for 2 weeks (Fig 2B) After one week, there was a significant reduction in α-SMA, EDA-fibronectin and calponin with a reduction in size and aspect ratio as depicted in Fig 1. A small reduction in the expression of myofibroblastic markers was seen by incubating for another week.

Culturing VICs in DMEM on fibronectin or collagen did not reduce the myofibroblastic differentiation (Fig 2C).

### DMEM promotes an activated VIC phenotype

**Fig 3:**
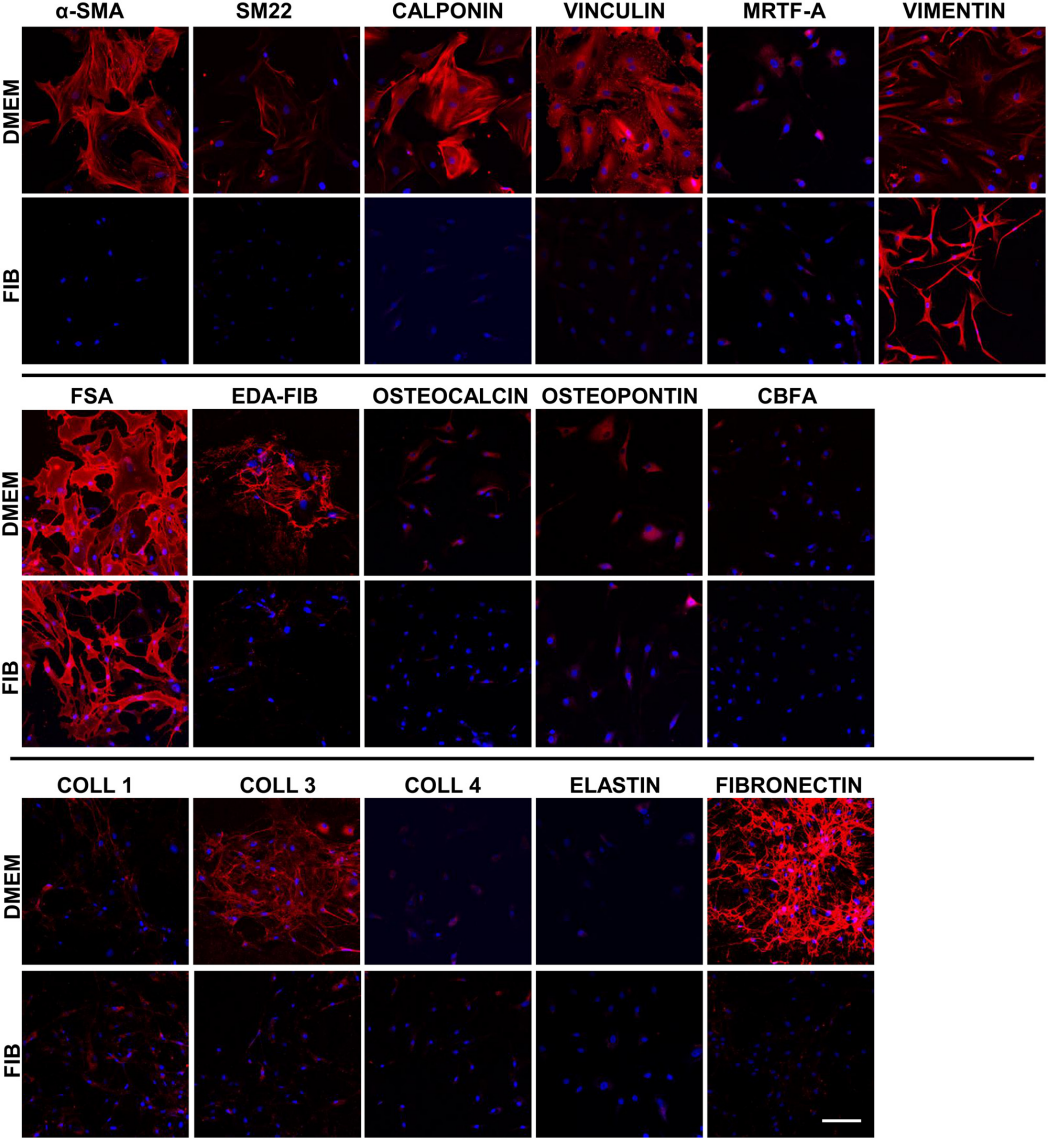
Immunostaining of VICs in DMEM and FIB media. **EDA-FIB, EDA-splice variant of fibronectin; coll, collagen**. Data labelled as FIB is from VICs that were switched to fibroblast media for 2 weeks prior to analysis. Scale bar represents 50μm.

VICs cultured in the two different media were stained using antibodies against a range of markers to include cytoskeletal, osteogenic and extracellular matrix proteins (Fig 3). Vimentin is an intermediate filament and a marker of mesenchymal cells and the majority of the cells of the valve express vimentin in situ. In culture, the VICs retain this expression however as the morphology of the VICs is different in the two media, long vimentin filaments can be discerned in the body of the VICs in DMEM whereas in the fibroblast media, the extensions are fine and compact and thus giving the impression of stronger staining.

When human VICs are isolated from the leaflet and cultured on plastic in standard DMEM, 56.9 ± 8.9% differentiate into myofibroblasts which express α-SMA [10]. This difference can clearly be seen in Fig 3 where the myofibroblastic VICs have strong staining for α-SMA in stress fibres spanning the cell as opposed to the weak, diffuse staining of cells in fibroblast media, with no visible stress fibres within the cells. There was a spectrum of staining patterns for α-SMA in DMEM; the majority of the VICs demonstrated strong stress fibre staining but this was accompanied by varying intensities in staining by the VICs indicating varying degrees of differentiation of the fibroblastic phenotype into the myofibroblast phenotype.

EDA-fibronectin is a splice variant of fibronectin which is specifically expressed in myofibroblasts. EDA-fibronectin was clearly seen in the majority of cells in DMEM to varying degrees indicating a spectrum of differentiation of the VICs into myofibroblasts. This marker was very weakly expressed by some VICs in fibroblast media.

The pattern of staining by vinculin (green) was markedly different in the two media (Fig 3). Vinculin stains focal adhesions and these could be clearly discerned in VICs cultured in DMEM. VICs cultured in fibroblast media showed diffuse, homogeneous staining by vinculin indicating very small focal adhesions. There was some co-localisation of vinculin and phalloidin (red) in VICs cultured in DMEM but not in VICs cultured in fibroblast media.

There appeared to be no difference in fibroblast surface antigen (FSA) staining (CD90) between the two media. There is a basal expression of markers of calcification such as cbfa, osteopontin and osteocalcin by VICs in DMEM and these were reduced in fibroblast media but not quantitated. There was no sign of calcification at any of the time points assessed.

The expression of fibronectin was markedly decreased in fibroblast media with fewer and thinner fibronectin fibres however the expression of elastin and collagen 1 and 4 did not change between the two media. The expression of collagen 3 was reduced in fibroblast media.

The expression patterns may be masked by the different aspect ratios and size of VICs in the 2 media hence to normalise this expression, Western blotting was performed with equal loading of protein and expression was normalised to GAPDH.

### Protein Analysis demonstrates a reduction in α-SMA

**Fig 4:**
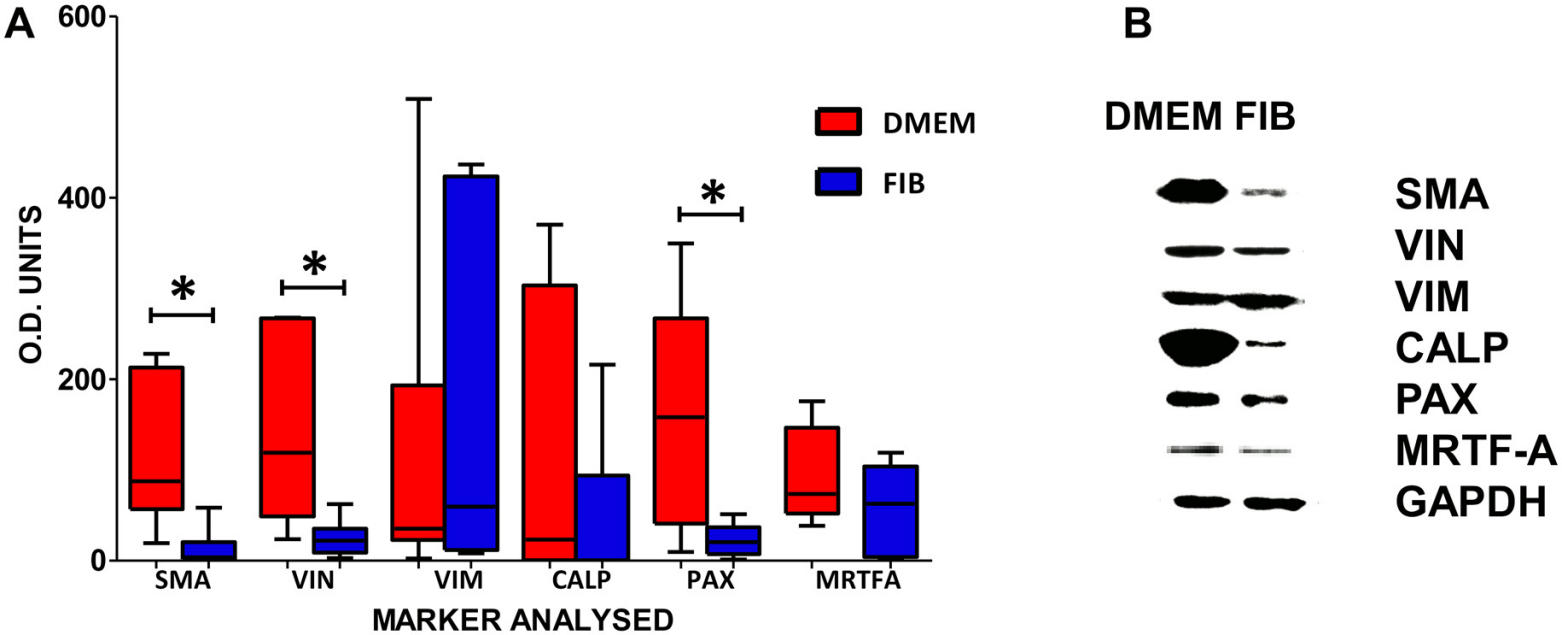
Graph showing the median levels of protein expression in VICs treated in DMEM and FIB as assessed by Western blotting (A) and representative Western blots (B). Data labelled as FIB is from VICs that were switched to fibroblast media for 2 weeks prior to analysis. * p < 0.005.

Paired isolates were maintained in DMEM and fibroblast media separately for 2 weeks and assessed for levels of protein expression by Western blotting. There was a significant reduction in the expression of α-SMA, (DMEM, median = 87.58; IQR = 56.72–213.0; FIB, median = 3.80; IQR = 1.7–20.6; P<0.005), vinculin (DMEM,median = 119.0; IQR = 48.94–267.2; FIB, median = 22.10; IQR = 8.94–35.21; P<0.01) and paxillin (DMEM, median = 158.4; IQR = 40.66–267.5; FIB, median = 20.78; IQR = 7.35–36.96; P<0.007) in VICs treated with fibroblast media (Fig 4). The EDA-fibronectin antibody was unsuitable for Western analysis as were the collagen antibodies.

### Proliferative capacity is reduced in fibroblast media

**Fig 5:**
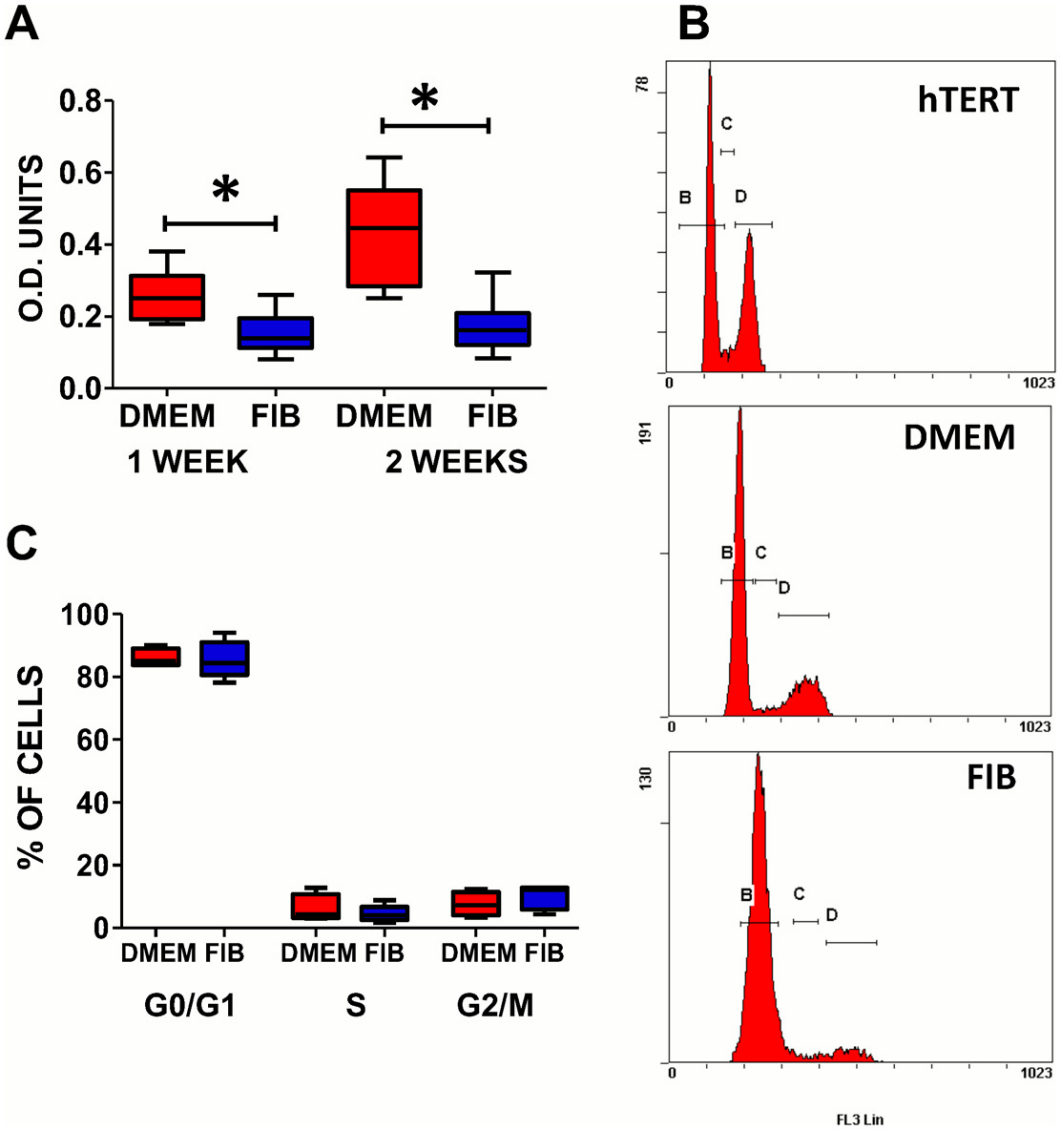
**Graph showing the proliferation of VICs in DMEM and FIB at 1 and 2 weeks (A)**. FACS histograms showing cell cycle analysis in hTert cells (positive control), DMEM and FIB treated cells (B) and graph showing the median levels of cells in the different phases of the cell cycle (C). Data labelled as FIB is from VICs that were switched to fibroblast media for 2 weeks prior to analysis.

VICs cultured in fibroblast media demonstrated a significantly reduced level of proliferation compared to those in DMEM at weeks 1 (DMEM median = 0.25; IQR = 0.19–0.31 and FIB median = 0.14; IQR = 0.11–0.19) and week 2 (DMEM median = 0.44; IQR = 0.28–0.55 and FIB median = 0.16; IQR = 0.12–0.21) (p<0.05) (Fig 5a). Cell cycle analysis of cells in both media did not show a significant difference in the percentage of cells in the different phases of the cell cycle (Fig 5B and 5C).

### VICs in DMEM demonstrate increased contraction and focal adhesions

**Fig 6:**
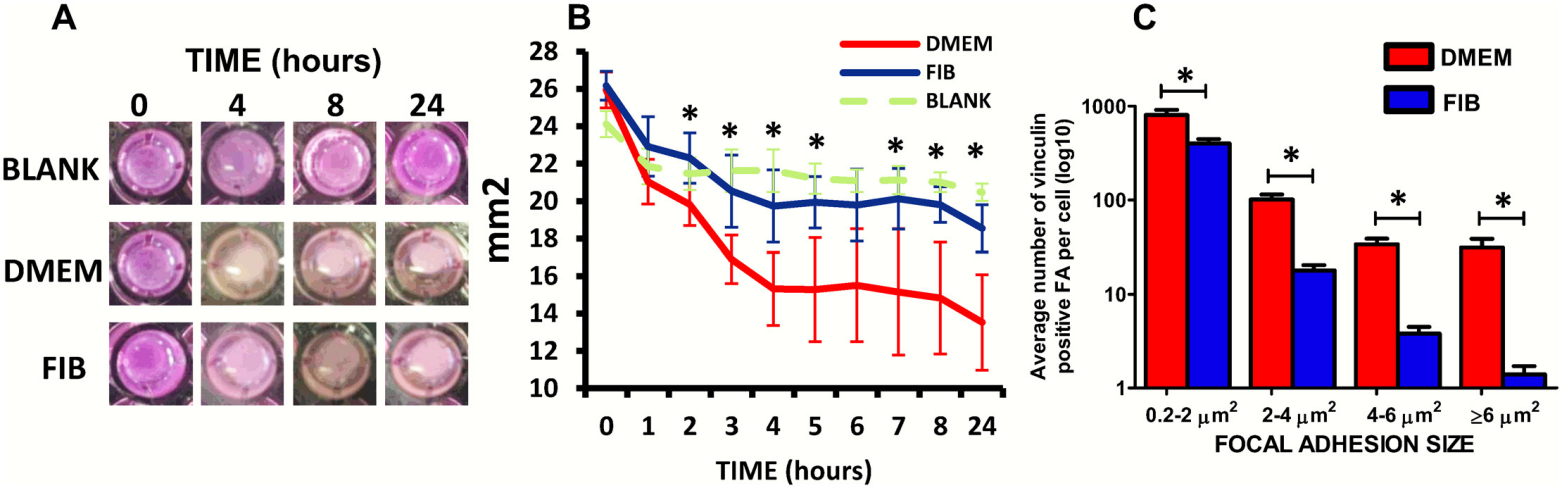
**Collagen gels showing differing degrees of contraction in DMEM and FIB media (A)**. Graph showing the temporal pattern of gel contraction by VICs in the 2 media (B) and graph showing the average number of different sized focal adhesions in the two media (C). Data labelled as FIB is from VICs that were switched to fibroblast media for 2 weeks prior to analysis.

VICs in both media had the capacity to contract collagen gels (Fig 6A) however VICs cultured in DMEM demonstrated significantly increased contraction of collagen gels (24%) compared to VICs in fibroblast media (15%) after 2 hours of releasing them from the well (p<0.01) (Fig 6B). After 24 hours, contraction remained significantly higher in the DMEM media with a 48.0% reduction in gel surface area compared to a 29.2% in reduction in fibroblast media (p<0.01).

This difference in contraction was attributed to the significantly larger focal adhesions of each size in DMEM compared to fibroblast media with a significantly greater number of supermature (>6μm) focal adhesions in DMEM (p<0.001) (Fig 6C).

### Ultrastructural differences of VICs in DMEM and fibroblast media

**Fig 7:**
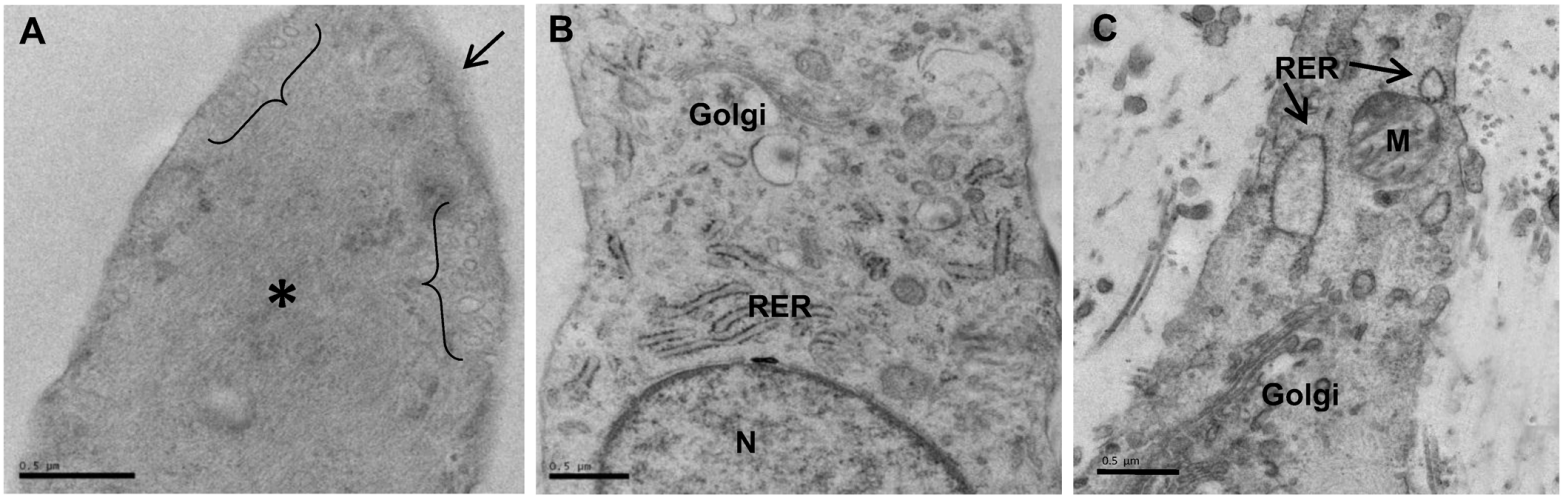
**Electron micrographs of VICs cultured in DMEM showing myofibroblast characteristics such as pinocytotic vesicles (brackets)**, abundant cytoplasmic myofibres (*) and incomplete external lamina (arrow) (A). VICs cultured in fibroblast media (B) and VICs in native aortic valve (C) showing abundant RER, golgi stacks and lack of myofibroblastic characteristics. Data in (B) is from VICs that were switched to fibroblast media for 2 weeks prior to analysis. Scale bar represents 0.5μm.

By electron microscopy, VICs in DMEM showed abundant myofilaments in their cytoplasm together with an incomplete basal lamina and membranous pinocytotic vesicles (Fig 7A). VICs in fibroblast media (Fig 7B) showed ultrastructural features similar to the native VICs (Fig 7C) with golgi stacks and RER and an absence of myofibroblastic features.

### VICs in DMEM demonstrate increased migratory ability

**Fig 8:**
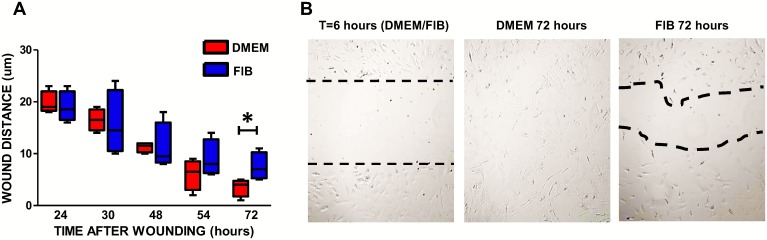
**Graph showing the migratory ability of VICs in DMEM and fibroblast media (A)**. Representative images of the wound at various time points after wounding (B). At 6 hours post-wounding, wound edges in both media were similar in lack of migration. Data labelled as FIB is from VICs that were switched to fibroblast media for 2 weeks prior to analysis. *p<0.01.

VICs in both media showed no difference in their ability to initially migrate into a wounded area, however by 54 hours post wounding, VICs in DMEM migrated faster than those in fibroblast media and had closed the wound by 72 hours. There was a significant difference in the distance migrated at 72 hours between VICs in DMEM (median distance = 4μm; IQR = 1.75–4.75) and VICs in fibroblast media (median distance = 7.0μm; IQR = 5.25–10.25) (p<0.01) (Fig 8).

## Discussion

VICs have been ascribed with a fibroblastic phenotype in situ due to the absence of markers specific to other cell types, their morphology, their adherence to plastic and their ability to synthesise extracellular matrix proteins. To our knowledge there is no specific marker for fibroblasts however they share a number of markers with other mesenchymal cells[13]. In culture, it has long been known that VICs are a heterogeneous population exhibiting spindly and cuboidal phenotypes along a spectrum of fibroblastic to myofibroblastic differentiation with secretory and contractile properties respectively[7, 12, 14]. This heterogeneous population in vitro does not emulate that of the healthy native valve cell population.

The majority of VICs are fibroblastic with a small population of smooth muscle cells within the base of the ventricularis[2]. These fibroblastic VICs do not express α-SMA or other markers of myofibroblastic differentiation in situ but rapidly acquire this expression in vitro, within 5–10 days. When first plated in culture, VICs are small compact cells with few filopodia. However after 10 days in standard culture media, a large proportion of VICs acquire a much larger, spread, rhomboid morphology. This is accompanied with the neo-expression of α-SMA stress fibres and markers of myofibroblastic differentiation. The expression of α-SMA is considered the basis for the designation of the term”myofibroblast” to the VICs, however this term encompasses many more features than simply the expression of α-SMA. We show, for the first time, the early expression of EDA-fibronectin (a splice variant of fibronectin specifically expressed by myofibroblasts) and SM22 by VICs cultured in classical DMEM. These myofibroblastic characteristics are all correlated with the generation of contractile force[29] and were significantly reduced using this fibroblast media.

Supermature focal adhesions are a hallmark of myofibroblasts and these were significantly reduced in fibroblast media. The absence of a statistically significant difference in the expression of vinculin and paxillin by Western blotting is explained by the huge intrinsic variability observed between patients. A reduction in the protein levels after fibroblast media treatment was systematically observed for the seven valve isolates, however the initial level of expression of focal adhesion proteins was highly variable. This is explained by the variability in the origin of the samples and in their age, gender, ethnicity etc., which reflects different genetic predispositions and possibly undiagnosed pathologies. Indeed with advancing age, risk factors increase for the development for calcific aortic stenosis and older patients may have a propensity for a greater degree of differentiation towards the pathological myofibroblastic phenotype.

DMEM supplemented with 10% fetal calf serum is used as a standard culture medium[2, 26–28] and together with in vitro culture on tissue culture plastic, results in the differentiation of the native VICs to a myofibroblastic phenotype to varying degrees within each isolate. In humans, we have reported that VIC differentiation occurs in approx. 56% of VIC cultures[10] based on strong α-SMA staining however this can go as high as 100% in porcine VIC cultures with increasing passage number (our unpublished data). However, based on SM22 and EDA-fibronectin expression, VICs cultured in DMEM are over 90% myofibroblastic. The variability in α-SMA positivity in culture is a result of the native VICs being a heterogeneous cell population with varying expression profiles. This variability may arise depending on cell-cell and cell-ECM contacts e.g. FGF2 binds heparin and heparan sulfate proteoglycans have been demonstrated to enhance and inhibit FGF2 activity [30]. The supplementation with 10% serum is a factor in this differentiation as it contains a multitude of growth factors, possibly including transforming growth factors (TGFs) and bone morphogenetic proteins (BMPs), hence a reduction in serum was utilised. Insulin was used as a mitogenic factor to enhance growth and survival[25]. It signals via canonical phosphoinositide 3-kinase/Akt and Ras/MAP kinase pathways [31]. The rigidity of the substrate is of central importance for induction of the phenotypic switch hence culture on tissue culture plastic which has a high elastic modulus proffers mechanical cues for myofibroblastic differentiation. Using the cocktail of FGF2, insulin and low serum concentration, we have been able to de-differentiate the in vitro myofibroblastic phenotype, to a phenotype more reminiscent of the native valve.

One of the major changes in fibroblast media was the significant reduction in α-SMA as shown by Western blotting and immunocytochemistry which was clearly discerned by the loss of stress fibres and the reduction in size of the cells. This result is consistent with those of Cushing et al[20] who demonstrated a decrease in α-SMA expression after 48hours of treatment with 10ng/ml FGF2. This was accompanied by the repression of Smad activity and their nuclear localisation. However, cell confluence seems to have a major impact on the basal expression level of α-SMA with sub-confluent cultures expressing more α-SMA[21].

This change in phenotype after treatment with fibroblast media is accompanied with a significantly lower proliferative rate. It was for this reason that VICs were cultured in DMEM initially to obtain sufficient numbers of VICs for investigation and then dedifferentiated with FIB media. For protocols requiring few cells, VICs were directly cultured in FIB media. Others have shown that optimum growth of VICs occurred at 15 to 20% serum concentrations [12], however this also modulates the phenotype of VICs. Cell proliferation has been assessed in semi-lunar and atrioventricular valves and the proliferation index was highest in developing endocardial cushions but absent in adult mouse valves [32]. The expression of Ki-67 was also shown to be significantly lower in adult valves, reducing by 90%, compared to that of the proliferative index of fetal valves[6]. Our unpublished data also indicates that a very low percentage of normal human VICs in valve leaflets are proliferative as indicated by the lack of expression of Ki67 and PCNA.

Migratory properties of the VICs treated with fibroblast media were significantly reduced. This result is associated with the reduction in focal adhesion proteins, vinculin and paxillin. Cells have been shown to produce greater migration forces when vinculin is recruited to the adhesion site [33] hence the myofibroblastic VICs have a greater migratory ability.

We demonstrate that this fibroblast media is able to reduce the expression of myocardin-related transcription factor–A (MRTF-A). The expression of MRTF-A is key in the differentiation of fibroblasts to myofibroblasts. We have previously shown that MRTF-A is expressed only by the small number of smooth muscle cells in the base of the ventricularis[2] and not by the VICs per se. In culture, a variable small number of VICs weakly express MRTF-A and MRTF-B (data not shown). Crider et al[34] demonstrated that TGF-β1 mediates myofibroblast differentiation and the expression of a contractile gene program through the actions of the MRTFs resulting in the increased expression of α-SMA, calponin, vinculin and SM-22. Moreover, inactivation of MRTF-A in myofibroblasts led to a reduction in the number and size of focal adhesions and a decrease in contractile potential. The transcriptional activity of MRTFs is regulated by actin dynamics[35, 36]. They are sequestered in the cytoplasm by G-actin and maintained transcriptionally inactive. The development of tension in the fibroblast, along with TGFβ1, promotes F-actin assembly[37] causing nuclear translocation of MRTF-A in a Rho-Rho-kinase (ROCK) dependent manner[38]. This leads to activation of the MRTF/SRF complex and the expression of α-SMA and other smooth muscle specific cytoskeletal proteins [39]. Targeting MRTF may provide a means to maintain VICs in their native state and to dedifferetiate activated myofibroblasts in diseased valves.

We have shown that normal human VICs and valve endothelial cells express FGF2 and this expression was increased in both cell types of calcified valves. As VICs in clacified valves are undergoing pathological differentiation, this increased expression may be an attempt to maintain the VICs in their native state. In healthy valve leaflets, this autocrine and/or paracrine expression of FGF2 may be pivotal in controlling the phenotype of valvular cells. However cell growth after treatment with this FGF2–containing media inhibited proliferation of human VICs. There are 3 major signal transduction pathways of FGF, including PLC/PKC, PI3K/Akt and Ras-Raf-Mek-MAP kinase [22, 40]. FGF2 is also able to moderate TGF-mediated downstream signalling however data from these studies are conflicting with FGF2 preventing TGFβ1-mediated Smad expression[20] and promoting TGFβ/Smad signalling[21]. An additional benefit may be gained by the addition of heparin in combination with FGF2 as this has been shown to cause a reversal of the myofibroblast phenotype[41].

FGF-2 has been shown to promote cell proliferation in various cell types[41], including porcine aortic VICs [12], osteoblasts, fibroblastic cells, mesenchymal cells[42] and vascular endothelial cells[30]. In contrast, others have not reported reduced cell proliferation and this may be due to the short duration of treatment with bFGF (up to 4 days) in previous studies[12, 20]. The reduced proliferation here, compared to standard DMEM media containing 10% FCS, by the fibroblast media is the result of the combination of bFGF, insulin and reduced serum.

We cannot discount that the standard media selects for specific sub-populations, it does differentiate over 90% of the VIC fibroblasts to vastly varying degrees of myofibroblastic differentiation. We have assessed osteogenic potential of VICs grown in both media and they are able to differentiate with the expression of alkaline phosphatase, alizarin red, von Kossa detected immunohistochemically and immunochemically using antibodies to osteogenic markers. A subpopulation of VICs undergo this differentiation and we do not know which human VIC subpopulation this is.

Our findings have major implications: The use of standard culture techniques to expand VICs is not appropriate if an understanding of how VICs function *in vivo* is sought. Additionally rigid 2D culture dishes promote their differentiation and as such, softer matrices and/or 3D matrices may be more suited [43]. Data from studies utilising cultured VICs that were attributed to “fibroblasts” without a thorough phenotypic and functional identification are most likely to have utilised predominantly myofibroblasts (in the case of porcine data) or at least a heterogeneous population of cells ranging from fibroblasts to protomyofibroblasts to fully differentiated myofibroblasts, in the case of human studies.

In conclusion, we have demonstrated that supplementation with FGF2 and insulin together with reduced serum in the media is able to maintain human VICs in a state more akin to their native phenotype and also to dedifferentiate and decrease the degree of differentiation of the cultured, predominantly myofibroblastic VICs toward the native VIC phenotype in vitro. We report a decrease in molecular markers including cytoskeletal proteins, focal adhesion complexes, a lower proliferative rate and changes in cell morphology and behaviour representative of a fibroblastic cell. Taking these observations together, this study demonstrates the potential of this FIB formulation to maintain human VICs in a state more representative of the native VIC phenotype. This formulation warrants testing for other fibroblasts and may prove useful for maintaining and culturing isolated fibroblasts in their native state. This provides a better model to study VIC biology, differentiation and mechanisms involved in valve disease and for obtaining specific insights into phenotypic and functional differences between the two cell types as well as their use in valve tissue engineering.
